# Molecular Characterization and Developing a Point-of-Need Molecular Test for Diagnosis of Bovine Papillomavirus (BPV) Type 1 in Cattle from Egypt

**DOI:** 10.3390/ani10101929

**Published:** 2020-10-21

**Authors:** Mohamed El-Tholoth, Michael G. Mauk, Yasser F. Elnaker, Samah M. Mosad, Amin Tahoun, Mohamed W. El-Sherif, Maha S. Lokman, Rami B. Kassab, Ahmed Abdelsadik, Ayman A. Saleh, Ehab Kotb Elmahallawy

**Affiliations:** 1 Department of Virology, Faculty of Veterinary Medicine, Mansoura University, Mansoura 35516, Egypt; tholothvirol@mans.edu.eg (M.E.-T.); dr.sama786@yahoo.com (S.M.M.); 2 Department of Mechanical Engineering and Applied Mechanics, University of Pennsylvania, Philadelphia, PA 19104, USA; mmauk@seas.upenn.edu; 3 Health Sciences Division, Veterinary Sciences Program, Al Ain Men’s Campus, Higher Colleges of Technology, Al Ain 17155, UAE; 4 Department of Animal Medicine (Infectious Diseases), Faculty of Veterinary Medicine, The New Valley University, El-Karga 72511, New Valley, Egypt; yasserelnaker@yahoo.com; 5 Department of Animal Medicine, Faculty of Veterinary Medicine, Kafrelshkh University, Kafrelsheikh 33511, Egypt; amin12_veta@yahoo.com; 6 Department of Surgery, Anesthesiology and Radiology, Faculty of Veterinary Medicine, The New Valley University, El-Karga 72511, New Valley, Egypt; drmwt@hotmail.com; 7 Biology Department, College of Science and Humanities, Prince Sattam bin Abdul Aziz University, Alkharj 11942, Saudi Arabia; ms.hussein@psau.edu.sa; 8 Department of Zoology and Entomology, Faculty of Science, Helwan University, 11795 Cairo, Egypt; rami.kassap@yahoo.com; 9 Department of Biology, Faculty of Science and Arts, Al Baha University, Almakhwah, Al Baha 1988, Saudi Arabia; 10 Zoology Department, Faculty of Science, Aswan University, Aswan 81528, Egypt; aabdelsadik@aswu.edu.eg; 11 Department of Animal Wealth Development, Genetics and Genetic Engineering, Faculty of Veterinary Medicine, Zagazig University, Zagazig 44519, Egypt; lateefsaleh@yahoo.com; 12 Department of Biomedical Sciences, University of Leon, 24071 León, Spain; 13 Department of Zoonoses, Faculty of Veterinary Medicine, Sohag University, Sohag 82524, Egypt

**Keywords:** bovine papillomavirus, cattle, Egypt, nucleic acid lateral flow immunoassay, PCR

## Abstract

**Simple Summary:**

Bovine papillomatosis is a disease caused by bovine papillomavirus (BPV), which is a diverse group of oncogenic viruses that challenge cattle industry, resulting in significant economic losses. The present study investigated the occurrence of bovine papillomatosis among cattle (*n* = 308) with cutaneous warts on the head and neck from New valley Province, Egypt through molecular detection of BPV-1, -2, -4, -5, and -10. The work also involved a phylogenetic analysis of the positive samples for detection of the genetic relatedness of the virus. Interestingly, BPV-1 DNA was detected in 84.6% of the collected samples. Furthermore, the study included the development of an isothermal nucleic acid amplification test, which is a field test combining molecular and lateral flow immunoassays for point-of-need testing appropriate for veterinary use in resource-limited settings. Collectively, our study provided interesting data related to the combined use of molecular and immunoassays methods in the detection of the virus besides better understanding the genetic relatedness of the circulating genotypes of BPV-1 in Egypt. Our study suggested further research to explore more about the other genotypes of BPV in the Egyptian environment that could be helpful for the implementation of control strategies for combating this disease.

**Abstract:**

Bovine papillomatosis is a viral disease of cattle causing cutaneous warts. A diagnosis of this viral infection is very mandatory for combating the resulting economic losses. Given the limited data available about bovine papillomavirus (BPV) in Egypt, the present study involved the molecular diagnosis of bovine papillomavirus type-1 (BPV-1), -2, -4, -5, and -10 in cattle presenting cutaneous warts on the head and neck from New Valley Province, Egypt. The phylogenetic analysis of the detected types of BPV was also performed, followed by developing a point-of-need molecular assay for the rapid identification of identified BPV types. In this regard, a total of 308 cattle from private farms in Egypt were clinically examined, of which 13 animals presented cutaneous warts due to suspected BPV infection. The symptomatic animals were treated surgically, and biopsies from skin lesions were collected for BPV-1, -2, -4, -5, and -10 molecular identification using polymerase chain reaction (PCR). The presence of BPV-1 DNA was confirmed in 11 collected samples (84.6%), while BPV-2, -4, -5, and -10 were not detected. Sequencing of the PCR products suggested the Egyptian virus is closely related to BPV found in India. An isothermal nucleic acid amplification test (NAAT) with labeled primers specific for the BPV-1 L1 gene sequence, and based on recombinase polymerase amplification (RPA), in combination with a lateral flow strip assay for the detection of RPA products, was developed and tested. The point-of-need molecular assay demonstrated a diagnostic utility comparable to PCR-based testing. Taken together, the present study provides interesting molecular data related to the occurrence of BPV-1 in Egypt and reveals the genetic relatedness of the Egyptian BPV-1 with BPV-1 found in buffalo in India. In addition, a simple, low-cost combined test was also validated for diagnosis of the infection. The present study suggests the necessity of future investigations about the circulating strains of the virus among the cattle in Egypt to assess their genetic relatedness and better understand the epidemiological pattern of the disease.

## 1. Introduction

Papillomaviruses are small nonenveloped viruses within the *Papillomaviridae* family with icosahedral symmetry, 55 to 60 nm in diameter, with a double-stranded DNA (dsDNA) genome and approximately 8-kilobase pairs in length [1,2]. The replication of these viruses occurs in the nuclei of squamous epithelial cells, and these viruses exhibit tropism to skin and mucosal tissues, causing benign and malignant tumors that replicate in the nuclei of squamous epithelial cells [3,4,5,6]. This group of viruses constitutes a wide range of DNA viruses that are found in mammals, birds, reptiles, and human beings [7]. In accordance with its occurrence in animals, the virus was identified in many domestic species, including bovine, ovine, swine, felines, and canines [8]. The resulting virus got its name based on the species from which the virus was characterized, as in case of bovine-named bovine papillomaviruses (BPVs) [8]. To our knowledge, twenty-six types of BPV have been described; 23 of them are grouped into five genera, with three types still unclassified [9]. The Deltapapillomavirus has four types: BPV-1, BPV-2, BPV-13, and BPV-14. The Xipapillomavirus genus has two species: Xipapillomavirus 1 (BPV-3, BPV-4, BPV-6, BVP-9, BPV-10, BPV-11, and BPV-15) and Xipapillomavirus 2 (BPV-12). The other two genus are Epsilonpapillomavirus 1 (BPV-5 and BPV-8) and Dyoxypapillomavirus 1 (BPV-7) [1,10,11,12,13]. Lastly, two recently described types (BPV-17) and BPV-20) are still unclassified as species. Taken into account, BPVs are generally species-specific; however, BPV-1, BPV-2, and BPV-13 can infect both cattle and equids [14,15,16]. Bovine papillomatosis is the resulting viral disease characterized by cutaneous warts or papillomas that represent proliferative lesions ranging from small nodular lesions to large cauliflower warts that are often rough and spiny to the touch and gray to black in color [17]. The transmission of BPV between animals may occur due to contaminated milking, ear-marking, grooming equipment, and animals rubbing on contaminated objects, such as wire fences [17]. Venereal warts may be transmitted sexually [6,17]. The prevalence of BPV is high in calves and yearlings, although all ages can be infected [17]. Steers are less frequently affected than heifers [6,18]. Regarding its occurrence in Egypt, some previous reports involved limited clinical and epidemiological studies on BPV in Egypt [18,19], but little yet is known about its occurrence in Egypt.

The accurate detection of the infected cases represents one of the main strategies for controlling the virus. In this regards, a tentative diagnosis is based on the clinical signs, while a confirmative diagnosis relies on histopathology, electron microscopy of the specimens, immunohistochemistry, and molecular or nucleic acid-based tests, e.g., polymerase chain reaction (PCR) [20,21]. To the authors’ knowledge, there are no reports of in vitro cultivation of BPV [22,23]. Importantly, the development of a low-cost, minimally instrumented, simple-to-use method for the rapid (30 to 60 min) detection of BPV would facilitate and inform the implementation of control measures and help reduce its economic impact. This method would enable sample testing in the field (e.g., on the farm) or at remote testing sites close to the farmer. In addition, it would eliminate the need for sample transport to central laboratories and the consequent delay times of days or weeks between sample collections and test results. Taking into consideration molecular tests based on specific enzymatic amplification of part of the virus genome, combined with either real-time or subsequent (post-amplification) detection of the amplicon (amplification product), are many advantages that include the highest sensitivity and specificity among all diagnostics methods [21,24]. However, conventional implementations of such molecular tests require relatively expensive instruments, i.e., benchtop thermal cyclers that provide precise and rapid temperature cycling of the sample, and are typically limited to use in facilities with reliable electric power and trained technicians [25]. In the past decade, the advent of isothermal amplification methods that use constant temperature instead of thermal cycling have fostered dramatic simplifications in point-of-care molecular diagnostics systems [26]. In laboratory settings, the amplification process is assayed by real-time fluorescent monitoring or post-amplification gel or capillary electrophoresis. These are costly or inconvenient for use outside of the laboratory. Alternatively, lateral flow strips (also called LF immunoassays or immunochromatography) provide convenient and simple noninstrumented methods for the detection of amplicons and are especially amenable to field tests. They are of low cost, compact (palm size), do not require electricity, and have a long shelf life [27]. For the detection of nucleic acids, the amplification primers are conjugated with antigen labels that bind with capture and reporter antibodies on the strip. The amplification product is blotted on the strip, and the presence of the amplicon (positive test result) is indicated by a darkened test line (and control line), whereas a negative test should, in principle, be implied by changes in only the control line [28]. For isothermal amplification, recombinase polymerase amplification (RPA) has a lower incubation temperature (37 °C), requires only two primers, compared to four or six with Loop-mediated isothermal amplification (LAMP), and generally exhibits very rapid amplification (10 to 20 min). RPA has demonstrated a sensitivity comparable to PCR, and for this work, with regard to point-of-need testing, RPA would appear advantageous [26,29,30,31]. Furthermore, LF immunoassays have proven suitable for on-site detection and field use in developing countries [27]. Combined RPA and LF immunoassays have previously been described for veterinary use for infectious bronchitis virus and Newcastle disease virus, as well as influenza virus (H9 subtype) detection [28,32]. Given the above information, our study was focused on the molecular detection and phylogenetic analysis of BPV-1, -2, -4, -5, and -10 in cattle from New Valley Province, Egypt presenting cutaneous warts on the head and neck that indicate a likely BPV infection. Furthermore, the work involved development of a field test combining RPA and LF immunoassays for point-of-need molecular testing as appropriate for veterinary use in resource-limited settings.

## 2. Materials and Methods

### 2.1. Ethical Statement

The ethical approval was performed as described by the ethical standards of Veterinary Medicine, Mansoura University, Egypt, which complies with all relevant Egyptian legislations. Cattle owners gave consent orally, which is in harmony with ethical regulations of the nation.

### 2.2. Animals

**Figure 1:**
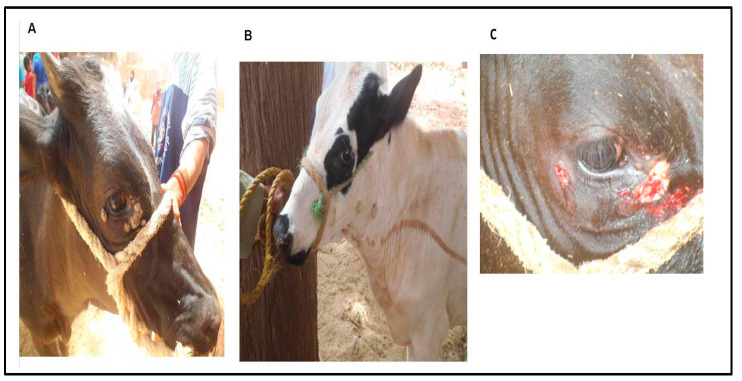
Typical cases of bovine papillomavirus (BPV) infection in cattle with macroscopic cutaneous warts around the eye (**A**), on the neck (**B**), and after surgical removal of cutaneous growths around the eye (**C**).

The study was carried out during the summer of 2016. A total number of 308 cattle (*Bos Taurus*) from private farms in New Valley Province, Egypt were examined (Table 1). Of which, 13 animals (9 females and 4 males, all aged 1 to 2 years) showed clinical signs consistent with BPV infection. Cattle over 2 years of age did not present any such clinical manifestations, and the history of these cases did not reveal the appearance of external signs of infection at any point in their lives. The clinically diseased cattle exhibited small, firm nodules on the head and cauliflower growths on the neck (Figure 1A,B). The skin lesions were grayish to black in color. The body temperatures of the infected cattle were within the normal range, and their appetites were normal.

### 2.3. Surgical Treatment of the Cutaneous Warts and Sampling

Diseased animals were sedated with 0.2 mg/kg xylazine 2% solution (Xyla-Ject, ADWIA Pharmaceuticals Co., Sharqia Governorate, Egypt) by intramuscular injection, and lidocaine HCL 2% (Hospira, Inc, 300 N Field Dr, Lake Forest, IL 60045, USA) was infiltrated around the cutaneous warts after preparation of the surgical site. Animals were restrained and typed before surgical excision. Excision of warts (Figure 1C) on the head and neck was performed by sharp scalpel until the blood oozed; then, hemorrhage was controlled. Povidone iodine 10% W/V skin solution (BETADINE^®^ antiseptic solution, El-Nile Co. for pharmaceuticals and chemical industries, Cairo, Egypt) was applied on the skin wounds to avoid secondary bacterial infection. All surgically treated cattle were injected with multivitamins (Elyoser Medicine Trading Co., Cairo, Egypt) by intramuscular injection. Recovery of the animals from clinical manifestation was checked 25 to 86 days post-treatment. Pieces of skin warts were collected after surgical excision. The samples were collected in bottles containing sterile saline for the molecular identification of BPV types 1, 2, 4, 5, and 10 by PCR. Skin samples from two apparently healthy cattle were involved as negative controls.

### 2.4. Skin Wart Sample Preparations

Samples from cutaneous warts were minced with sterile scissors and homogenized. The samples were then suspended in phosphate-buffered saline (20% w/v PBS) solution and centrifuged at 2000 rpm for 15 min, from which supernatants were stored at −20 °C for PCR tests.

### 2.5. Polymerase Chain Reaction (PCR)

Molecular identification of BPV-1, -2, -4, -5, and -10 in head and neck skin wart specimens was made with PCR. Oligonucleotide primers were synthesized for the amplification of the L1 gene of BPV-1 and -2, the E7 gene of BPV-4, and the E2 gene for BPV-5 and -10 according to protocols described elsewhere [33,34]. Primers were synthesized by Metabion International AG, Planegg, Germany and used at a 10-µM concentration. DNA extraction was carried out using a QIAamp^®^ MiniElute^®^ Virus Spin Kit (QIAGEN, GmbH, Hilden, Germany) based on the manufacturer’s instructions. Negative control skin samples were also involved. PCR amplification was done following a protocol described elsewhere [33], using a Thermo Scientific PCR Master Mix (Thermo Scientific, Waltham, MA, USA). The PCR conditions and time-temperature program was as follows: 95 °C for 10 min for initial melting, 30 cycles of 94 °C for 45 s (melting), 50 °C for 45 s (annealing), and 72 °C for 1 min (extension), followed by 72 °C for 7 min (final extension). Visualization of the PCR products by gel electrophoresis was performed as reported elsewhere [35].

### 2.6. PCR Product Sequencing and Analysis

Purification of PCR products from agarose gel was done using a QIAquick Gel Extraction kit (Qiagen Inc., Valencia, CA, USA). The ABI Prism BigDye™ Terminator v3.1 Cycle sequencing kit was used for DNA sequencing of the PCR amplicon using an ABI PRISM 3130x1 Genetic Analyzer (Life Technologies, Carlsbad, CA, USA). Analysis of the sequencing data was performed using ClustalW (http://www.ebi.ac.uk/Tools/msa/clustalw2/). The alignment *.aln output file was utilized for the neighbor-joining phylogenetic analysis, as well as divergence, and identity percent calculation was carried out via Mega software v5.2.2 (http://www.megasoftware.net/)

### 2.7. Developing Recombinase Polymerase Amplification-Nucleic Acid Lateral Flow Immunoassays (RPA-NALF) for Point-of-Need Molecular Identification of BPV

A point-of-need rapid molecular assay comprising an isothermal RPA amplification step with BPV-1-specific primers for the detection of conjugated amplicons with a lateral flow strip was developed to detect BPV-1.

#### 2.7.1. Oligonucleotide Primers for RPA

Primers were designed to amplify a 105-bp fragment based on the sequence of the virus gene (accession sequence MH543316) targeted for RPA amplification. The forward primer (5′CCTGATCCCAATCAATTTGC-3′) was labeled with DIG, and the reverse primer (5′-AGAGGCTGCCCTCTGGAC-3′) was labeled with Biotin. A BLAST search (http://www.ncbi.nlm.nih.gov) indicated no potential cross-hybridization of the primers with other bovine papillomaviruses, capripoxviruses, or bovine herpesvirus 2 (BHV2).

#### 2.7.2. BPV-1 RPA Amplification

The twistAmp™ Basic Kit (TwistDx Ltd., Cambridge, UK) was used for isothermal RPA. Briefly, 4.8 µl of forward and reverse primers (480 nM of each), 29.5-µl rehydration buffer, and 11.2 µl of nuclease-free water were added to RPA tubes provided in the kit and containing lyophilized enzyme and other reagents. Additionally, 2.5 µl of 280-nM/Mg acetate was placed on the inside of the tube lid, which was mixed on tube inversion. Finally, 2 µl of DNA from the sample was added to the tube. The tube was briefly centrifuged and placed in a water bath (38 °C) for 30 min. Nontemplate (no sample DNA) controls were also included. Samples with lumpy skin disease virus (LSDV) and sheep poxvirus (SPV) were used as a check for nonspecific amplification. All reactions were repeated three times.

#### 2.7.3. Visualization with Nucleic Acid Lateral Flow (NALF) Strip

Labeled RPA amplicons were assayed using Abington Health Lt (York, UK) PCRD lateral flow (nitrocellulose) strip immunoassay cassette, which can detect DIG/BIO-conjugated amplicons. Following RPA, 5 µl of RPA product and 70 µl of PCRD buffer were loaded into the PCRD cassette sample well, and the cassette was laid horizontally for at least five minutes. The DIG/Biotin amplicon bonded with colloidal carbon coated with anti-biotin detection antibodies (bonded with the biotin label). The carbon-conjugated amplicon migrated as the buffer wicked down the strip. The first test line on the nitrocellulose strip was striped with anti-DIG to capture carbon particles, which aggregated to darken the test line as a visual positive indicator of the amplicon. A second test line (striped with anti-FAM antibodies) was not used in this test. Further downstream, a control line was involved to capture excess carbon particles (not captured at the test lines) and as an indicator that the assay was working properly.

#### 2.7.4. The Limit of Detection (LOD)

Purified BPV DNA standard with 10^6^ viral genome copies/µL in Tris-EDTA buffer was used to estimate the minimum copies number of BPV-1 nucleic acid that can be identified via the RPA-NALF assay [36]. Serial dilutions (ten-fold) of purified BPV DNA were spiked into negative samples to estimate the LOD.

#### 2.7.5. BPV RPA-NALF Immunoassay Detection Performance

Thirteen clinical samples (*n* = 13) collected from suspected cattle and assayed by PCR, along with negative controls, were tested using the RPA-NALF immunoassay test described above.

## 3. Results

### 3.1. PCR Detection and Sequence Analysis

As mentioned above, PCR products from skin wart biopsies were visualized by gel electrophoresis. Out of 13 collected samples, 11 (eight female and three male) were positive for the BPV-1 L1 gene, with an amplicon of 301-bp size. BPV-2, -3, -5, and -10 were not detected, and the controls from healthy animals showed no PCR product. Accordingly, the prevalence of BPV-1 in our study population was 3.6% (11 out of 308). The results showed the infection rate more in females (2.6%) than male ones (1.0%). In accordance with sequence analysis, sequencing of the PCR product partial L1 gene showed 100% identity between our 11 positive specimens.

**Figure 2:**
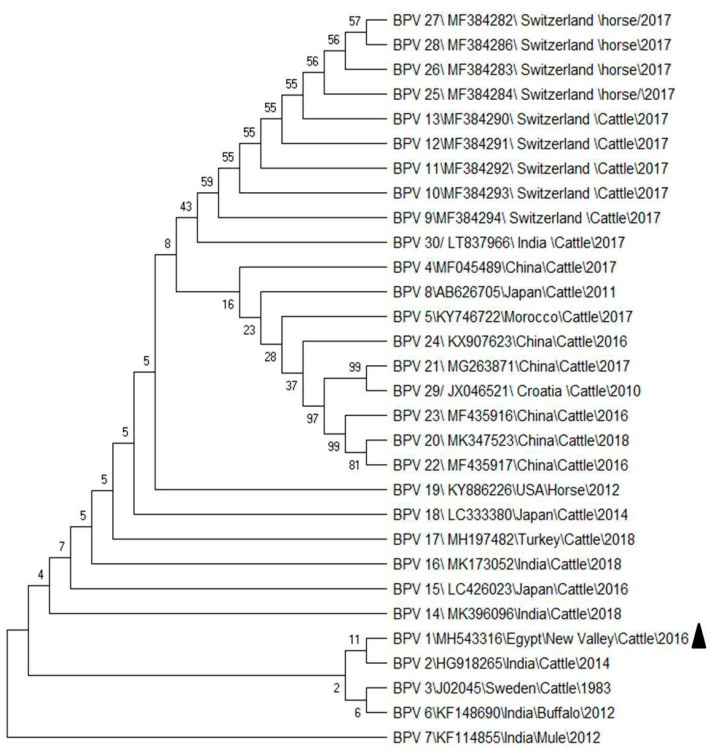
Phylogenetic tree of our bovine papillomavirus type-1 (BPV-1) from cattle with others BPVs that were taken from the GeneBank database based on L1 gene sequences. Numbers at the internal nodes represent the bootstrap probabilities (1000 replicates).

The sequence was submitted to GeneBank (accession: MH543316) and compared with similar sequences (Table 2) from China, India, Sweden, Morocco, Switzerland, Turkey, USA, Croatia, and Japan. The phylogenetic analysis (Figure 2) showed the Egyptian BPV in the same clade and closely related to Indian BPV from cattle that were identified in 2014 at both the DNA and protein sequences. The virus sequenced in the present work revealed 99.7% identity with the Indian virus (accession number HG918265).

### 3.2. RPA-NALF Assay Results

**Figure 3:**
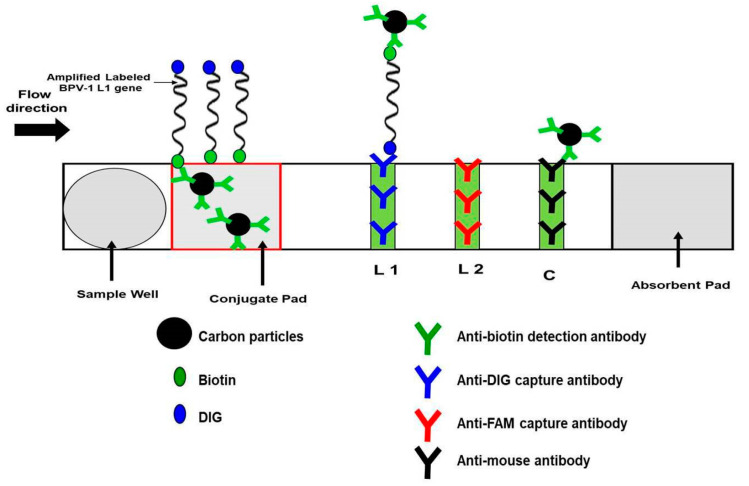
PCRD cassette. The carbon-conjugated biotin antibodies at the conjugate pad bind to biotin of the BPV-1 L1 gene amplicons and flow towards L1 and L2. L1 is lined with anti-DIG monoclonal antibodies to bind the labeled amplicons. L2 is decorated with anti-FAM monoclonal antibodies and will remain free from any carbon particles. C line is the control line that is lined with anti-mouse antibodies and will capture excess carbon-conjugated biotin.

**Figure 4:**
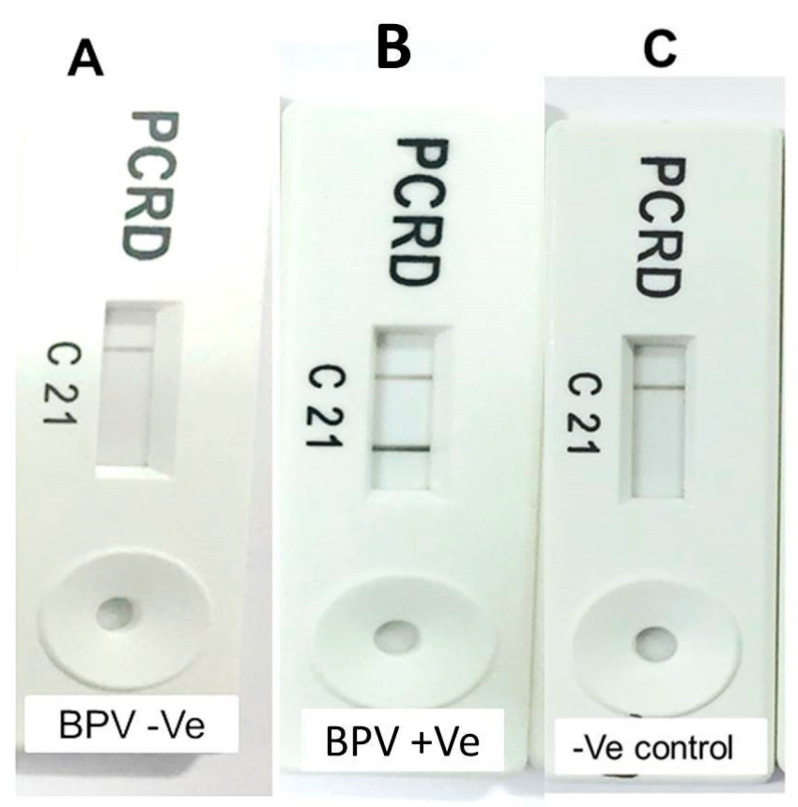
Results of recombinase polymerase amplification (RPA) amplicons identification after 30-min incubation at 38 °C using a PCRD cassette: (**A**) BPV assay revealed a negative reaction, (**B**) BPV assay revealed a positive reaction, and (**C**) negative result of the negative control.

As shown in Figure 3, PCRD cassettes were observed after incubation in a horizontal position for 5 min, with dark lines at the control line (C) and test line 1 (L1). Negative controls revealed dark lines only at the control line (C) (Figure 4). The LOD of our assay (based on the previously mentioned serial dilution tests) was approximately 100 viral genome copies per RPA reaction. RPA-NALF immunoassay was used to screen 13 samples for BPV, and it showed a 84.6% positivity rate, which is similar to that obtained by PCR.

## 4. Discussion

To the authors’ knowledge, BPV is associated with several clinical problems that might result in considerable economic losses in cattle production due to the resulting damage of infected cattle’s hides and dairy industries [37,38,39]. BPV can also affect the udders and teats of lactating cows, which might interfere with the suckling of young calves and milking of infected animals, besides predisposing the animals to secondary bacterial infection, which might result in mastitis [19,40]. Moreover, BPV infection causes gastrointestinal and bladder cancers in cattle, and further, the virus has been confirmed in the peripheral blood [41,42,43,44]. Despite the economic impact of BPV, there is insufficient information regarding the types of BPV circulating in Egypt. Interestingly, the present study reports BPV-1 (type 1) infection in cattle less than two years of age in New Valley Province. Furthermore, other BPV types (BVP-2, -4, -5, and -10) were not detected in wart specimens collected from the symptomatic cattle. Moreover, the phylogenetic analysis revealed that the Egyptian BPV is closely related to strains reported in India. The virus was molecularly identified based on the amplification and sequencing of L1 gene fragments. As shown, a lower prevalence (3.6%) of BPV-1 infection was found in this study than previous studies either at the national or international level [18,19,45,46]. This difference might be attributed to various factors, including differences in cattle management systems, sampling locations, and sample sizes [10,47,48]. In the present work, the higher infection rate of female cattle versus males may be due to the immunosuppression associated with pregnancy and lactation [49]. These observations are consistent with those described elsewhere [50]. In addition, the treatment regime in the current study showed that cattle recovered from 25 to 86 days post-surgical excision. This treatment was similar to that used previously for BP-infected cattle [18]. As depicted in our results, BPV-1 could be only detected in cattle less than two years of age, while examined animals over two years old did not reveal such clinical signs at the time of examination. The history of these older cases revealed that they did not show the external signs of the disease throughout their lives. The lack of infection in older animals may be due to more well-developed immune systems, and the absence of clinical signs in these animals when they were young could be due to their good health status or may be the absence of the source of infection at that time [3,7,10].

In accordance with its diagnosis, several previous studies documented the role played by the molecular methods in the detection and characterization of various strains of BPV [19,51,52,53,54]. The present study confirmed the occurrence of BP infection in Egypt and further demonstrated the typing of the causative virus by molecular methods. These methods offer many advantages over the immunological methods that require specific antibodies against strains of BPV that may not be readily available in developing countries such as Egypt [19,51,52,53,54]. The two PCR-negative samples of putative BPV specimens may be due to infection by other strains of BPV than those investigated in our study. Sequencing the L1 gene of BPV-1 revealed a close genetic relationship with BPV-1 found in India, suggesting a possible transmission of BPV from India to Egypt. Further studying of the genetic relationships and diversity among BPVs may identify other types of BPV that might circulate in Egypt and provide more information of their places of origin, patterns of spreading, or other causes of cattle warts. Furthermore, investigation of the virus using immunohistochemistry and histopathological methods would be interesting to gain a better understanding of bovine papillomatosis in Egypt

Timely control measures are essential to curtail BPV spread, and therefore, a second aim of the present work was to develop a simple, easy-to-use point-of-need molecular test appropriate for use outside of laboratories, which could provide test results in less than about 30 min. The current work also investigated a test comprising RPA available commercially as a tube-based reaction, with lyophilized reagents in combination with a commercially available NALF strip immunoassay cassette to visually detect amplification products, as demonstrated for the sensitive (LOD: 100 viral genome copies) detection of BPV in samples derived from cattle wart excisions. This test provides a minimally instrumented, simple-to-interpret, fast diagnostic for BPV infection appropriate for a limited-resource setting [55,56,57]. The development of nucleic acid amplification tests (NAATs) similar to that used in the present study enhances both the sensitivity and specificity of diagnostics of various infectious agents compared to simple immunoassays [58,59]. In addition, the isothermal RPA method obviates the need for an expensive thermal cycler unit as needed for PCR. Further development integrating nucleic acid extraction into simple tests will facilitate their wider use and convenience and improve reliability and performance [60].

## 5. Conclusions

The present findings concluded that BPV-1 was molecularly confirmed in cattle in Egypt using NAATs. The tests found no incidence of other BPV types (BPV-2, -4, -5, and -10) in cattle wart specimens. Our study revealed the close genetic relatedness of the Egyptian BPV-1 with BPV-1 found in buffalo in India. Taken into account, the control of BPV infection and mitigation of its economic impact can be facilitated by simple point-of-need tests. A simple low-cost combined RPA-NALF test was also developed and validated for the point-of-need molecular diagnostics of cattle suitable for use outside of laboratories. Further studies seem mandatory to investigate the other circulating strains of BPV in Egypt, combined with assessing the risk factors for BP in Egypt being warranted, especially comparing different cattle management systems.

## Figures and Tables

**Table 1:** Details of examined animals (*n* = 308) that showed clinical signs of bovine papillomatosis and positive result by bovine papillomavirus type-1-polymerase chain reaction (BPV-1-PCR).

Farm Number	Age Range (Year)	Total Number of Animals/Farm	Sex of Examined Animals		No, Sex, and Age of Animals Showed Clinical Signs and Positive Result by BPV-1-PCR	
			Female	Male		
**1**	1–4	15	10	5	0	
2	1–5	19	11	8	1 male (13 month)	
3	1–3	13	9	4	0	
4	1–5	21	13	8	1 Female (18 months) 1 male (24 month)	
5	1–5	16	10	6	0	
6	1–3	10	8	2	1 male (23 month)	
7	1–4	15	11	4	0	
8	1–3	18	12	6	0	
9	1–3	12	8	4	0	
10	1–5	14	10	4	1 Female (14 month)	
11	1–4	16	11	5	1 Female (13 month)	
12	1–5	15	10	5	0	
13	1–3	20	12	8	0	
14	1–4	14	10	4	1 Female (20 month)	
15	1–4	17	11	6	1 Female (15 month)	
16	1–5	10	8	2	0	
17	1–4	19	12	7	1 Female (14 month)	
18	1–5	19	13	6	0	
19	1–3	12	9	3	1 Female (17 month)	
20	1–5	13	9	4	1 Female (22 month)	
Total (%)		308 (100%)	207 (67.2%)	101 (32.8%)	11 (3.6%)	
					8 females (2.6%)	3 males (1.0%)

**Table 2:** Detailed information of L1 gene sequences of bovine papillomavirus (BPV) type-1 used in the present study.

Isolate Number	Country of Isolation	Year of Identification	Host Species	Accession Number
BPV 1	Egypt\New Valley Governorate (This study)	2016	Cattle	MH543316
BPV 2	India	2014	Cattle	HG918265
BPV 3	Sweden	1983	Cattle	J02045
BPV 4	China	2017	Cattle	MF045489
BPV 5	Morocco	2017	Cattle	KY746722
BPV 6	India	2012	Buffalo	KF148690
BPV 7	India	2012	Equine	KF114855
BPV 8	Japan	2011	Cattle	AB626705
BPV 9	Switzerland	2017	Cattle	MF384294
BPV 10	Switzerland	2017	Cattle	MF384293
BPV 11	Switzerland	2017	Cattle	MF384292
BPV 12	Switzerland	2017	Cattle	MF384291
BPV 13	Switzerland	2017	Cattle	MF384290
BPV 14	India	2018	Cattle	MK396096
BPV 15	Japan	2016	Cattle	LC426023
BPV 16	India	2018	Cattle	MK173052
BPV 17	Turkey	2018	Cattle	MH197482
BPV 18	Japan	2014	Cattle	LC333380
BPV 19	USA	2012	Equine	KY886226
BPV 20	China	2018	Cattle	MK347523
BPV 21	China	2017	Cattle	MG263871
BPV 22	China	2016	Cattle	MF435917
BPV 23	China	2016	Cattle	MF435916
BPV 24	China	2016	Cattle	KX907623
BPV 25	Switzerland	2017	Equine	MF384284
BPV 26	Switzerland	2017	Equine	MF384283
BPV 27	Switzerland	2017	Equine	MF384282
BPV 28	Switzerland	2017	Equine	MF384286
BPV 29	Croatia	2010	Cattle	JX046521
BPV 30	India	2017	Cattle	LT837966
